# Emergence of Resistant *Escherichia coli* Mutants in Microfluidic *On-Chip* Antibiotic Gradients

**DOI:** 10.3389/fmicb.2022.820738

**Published:** 2022-03-22

**Authors:** Krisztina Nagy, Barbara Dukic, Orsolya Hodula, Ágnes Ábrahám, Eszter Csákvári, László Dér, Miles T. Wetherington, Janneke Noorlag, Juan E. Keymer, Péter Galajda

**Affiliations:** ^1^Institute of Biophysics, Biological Research Centre, Szeged, Hungary; ^2^Department of Biotechnology, University of Szeged, Szeged, Hungary; ^3^Doctoral School of Multidisciplinary Medical Sciences, University of Szeged, Szeged, Hungary; ^4^Department of Natural Sciences and Technology, University of Aysén, Coyhaique, Chile

**Keywords:** antibiotic resistance, evolution, microfluidics, antibiotic gradients, spatial heterogeneity

## Abstract

Spatiotemporal structures and heterogeneities are common in natural habitats, yet their role in the evolution of antibiotic resistance is still to be uncovered. We applied a microfluidic gradient generator device to study the emergence of resistant bacteria in spatial ciprofloxacin gradients. We observed biofilm formation in regions with sub-inhibitory concentrations of antibiotics, which quickly expanded into the high antibiotic regions. In the absence of an explicit structure of the habitat, this multicellular formation led to a spatial structure of the population with local competition and limited migration. Therefore, such structures can function as amplifiers of selection and aid the spread of beneficial mutations. We found that the physical environment itself induces stress-related mutations that later prove beneficial when cells are exposed to antibiotics. This shift in function suggests that exaptation occurs in such experimental scenarios. The above two processes pave the way for the subsequent emergence of highly resistant specific mutations.

## Introduction

The driving force behind the research on antimicrobial resistance is twofold. On the one hand, it is an important topic in basic research that helps to answer fundamental questions about evolution. On the other hand, bacteria with antibiotic resistance pose a significant threat to human health, through which they affect society. There is a growing recognition to merge these seemingly separate efforts and apply the concepts of ecology and evolution in medicine (Andersson et al., 2020). There are, however, important but less studied ecological and evolutionary aspects of antibiotic resistance, such as the role of spatiotemporal antibiotic gradients. Chemical gradients are widespread in natural habitats (including the human body), in which gradient-dependent phenomena like chemotaxis occur. Microfluidic technologies provide the tools (Wang et al., 2017) to establish and control habitat gradients in space and time to effectively explore their role in evolutionary processes (Zhang et al., 2011; Hol et al., 2016; Alcalde et al., 2019, 2021; Deng et al., 2019).

Antibiotics have a concentration-dependent effect on bacterial cells (Bernier and Surette, 2013). While they are toxic at high concentrations, they may promote the emergence of persistent and resistant cells in the population at low concentrations (Andersson and Hughes, 2014). The challenge imposed by antibiotics may also induce various stress responses in bacteria, resulting in phenotypic adaptation and tolerance that may serve as the first step in the evolutionary pathway leading to resistance (Fridman et al., 2014; Hol et al., 2016; Levin-Reisman et al., 2017, 2019). Such resistant cells acquire mutations that help cope with antibiotics by increased efflux activities, enzymatic inactivation, target modification, and biofilm formation, among others (Walsh, 2000; Martinez et al., 2009; Blair et al., 2015).

The concentration-dependent effects of antibiotics suggest that the uneven spatial distribution of antibiotic agents may substantially impact the evolution of resistance. Indeed, theoretical studies indicate that antibiotic concentration gradients may accelerate the evolution of resistance (Greulich et al., 2012; Hermsen et al., 2012). Despite these theoretical predictions, few experimental studies involve spatially heterogeneous antibiotic concentrations. Zhang et al. (2011) observed fast evolution of resistance of *Escherichia coli* to ciprofloxacin within 10 h in a structured heterogeneous environment in a microfabricated device (Zhang et al., 2011). As suggested by theory, the structure of these microfluidics-based environments may act as selection amplifiers for the evolution of resistance (Tkadlec et al., 2021). Other experiments carried out on soft agar plates suggested that motility (surface-associated swarming behavior and swimming) may have an important role in the adaptation and evolution in structured environments (Lai et al., 2009; Butler et al., 2010; Baym et al., 2016; Ghaddar et al., 2018). Interestingly, a recent study comparing the effect of spatial concentration gradients of ciprofloxacin to homogeneous batch culture experiments on the development of antibiotic resistance suggests the contrary of the results mentioned above. Deng et al. (2019) observed that non-motile *E. coli* cells evolved much higher resistance against ciprofloxacin in batch experiments where antibiotic concentrations were increased in time.

Evolutionary pathways to antibiotic resistance depend not only on environmental structure (spatial and temporal) but bacterial lifestyle as well. Planktonic populations and biofilms of *Acinetobacter baumannii* gained different resistance levels to ciprofloxacin by acquiring various mutations that enhanced the levels of resistance via pleiotropic interactions. As spatial systems avoid clonal interference, a higher diversity of variants produces enhanced levels of resistance (Santos-Lopez et al., 2019).

Despite these important studies, a detailed, systematic experimental exploration of the effect of antibiotic concentration gradients on the evolution of resistance is needed. In particular, persistence, stress, and pleiotropy’s roles in the evolution of resistance in spatially distributed bacterial ecosystems need to be explored.

We aimed to shed light on the fundamental processes behind antibiotic resistance. In a microfluidic device, we exposed non-resistant motile *E. coli* bacteria to linear spatial concentration gradients of ciprofloxacin. We observed the cellular and population-level response to various gradients of ciprofloxacin for up to 72 h. We then performed genomic sequencing to identify key mutations leading to antibiotic resistance.

## Materials and Methods

### Bacterial Strains and Growth Conditions

The experiments were performed using the *E. coli* W3110 strain JEK1036 (Keymer et al., 2008) labeled with green fluorescent protein *(lacYZ:GFPmut2*). The mutation rate for this strain was measured to be in the order of 10^–10^ per bp per generation (Kishimoto et al., 2010). The expression of the fluorescent protein was induced by adding 1 mM isopropyl β-D-1-thiogalactopyranoside (IPTG) to the culture media. We used the same *E. coli* W3110 strain in co-culture experiments but labeled with red fluorescent protein (*lacYZ:mRFP*, known as JEK1037). The minimal inhibitory concentration (MIC) for ciprofloxacin was determined for these strains in 96-well plates, following the protocol described in Wiegand et al. (2008). The MIC was measured to be 16 ng/ml. The surface adhered biofilm forming ability of the wild-type strain and selected mutants was tested using the microtiter plate biofilm assay (Coffey and Anderson, 2014) with 24 h incubation at 30°C in antibiotic-free LB, and LB supplemented with 10 and 20 ng/ml ciprofloxacin.

Cells were taken from a −80°C glycerol stock and grown on solid (1.5%) LB agar plates. Before each microfluidic experiment single colonies were taken and grown overnight (16 h) in 3 ml lysogeny broth (LB) medium in a shaker incubator (30°C, 200 rpm). Overnight cultures were then diluted in a 1:1,000 ratio with LB medium containing 1 mM IPTG and grown to an optical density of 0.6 (measured at 600 nm). At that point, the bacterial suspension was further diluted to reach the desired cell density to inoculate the observation channel of the microfluidic device or the microwell plates for MIC measurements. Initial cell density in MIC measurements was 5 × 10^5^ cells/ml. Microfluidic devices were typically inoculated with 10^5^ cells (10^3^ cells in those cases where we visualized cell morphology and localized biofilm formation, as shown in Figures 2C,D). For the co-culture studies, we used mixed suspensions of the wild-type and the selected mutant strain (s10/16) in a 1:1 ratio to inoculate the microfluidic device.

### Microfluidic Device Setup

**FIGURE 1:**
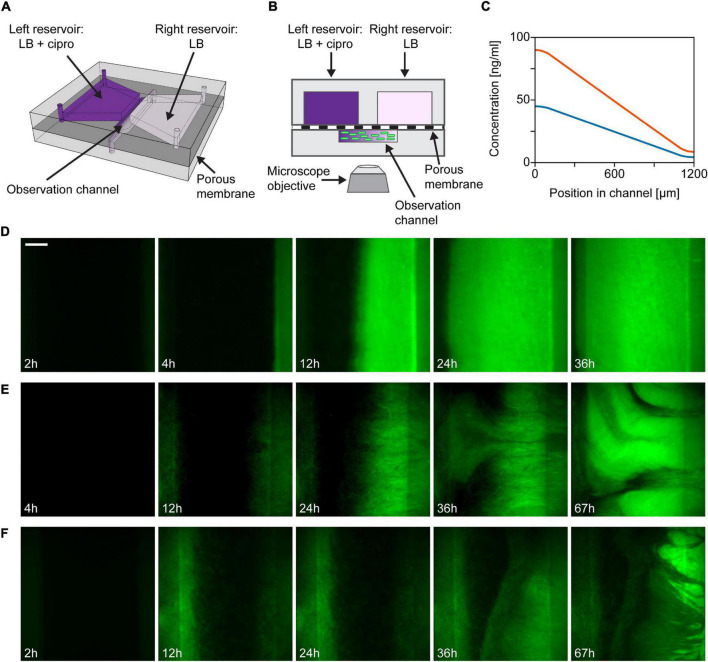
Outline of the gradient generator microfluidic setup and time-lapse images of a growing bacterial population within. **(A)** Schematic drawing of the microfluidic gradient generator device (not-to-scale). **(B)** Illustration of the cross-sectional view of the device (not-to-scale). **(C)** Theoretical profiles of ciprofloxacin concentration across the width of the observation channel in case of loading 48 ng/ml (3× MIC, blue curve) or 96 ng/ml (6× MIC, orange curve) antibiotic solution into the left reservoir. **(D–F)** Fluorescent images showing the distribution of bacteria across the observation channel (scale bar is 200 μm). Maximum ciprofloxacin concentrations (left side) are as follows: **(D)** 3× MIC; **(E)** 3× MIC; and **(F)** 6× MIC.

The design and fabrication of the microfluidic device have been described in detail in Nagy et al. (2015). Figure 1 shows the schematic representation of the device. Two reservoirs (∼50 μl each) and an observation channel (∼0.4 μl), molded in polydimethylsiloxane (Sylgard 184, The Dow Chemical Company, Midland, MI, United States) are situated on opposite sides of a porous membrane (pore size: 0.1 μm; Anodisc 47 mm, Whatman plc, Maidstone, United Kingdom). Each side of the observation channel overlaps with one of these reservoirs in a narrow region. Diffusion of molecules from one reservoir to the other through the membrane establishes a linear concentration gradient across the 1.2 mm width of the observation channel, where cells may be injected. The concentration of ciprofloxacin within the observation channel was calculated for a full 3D model with Comsol Multiphysics 4.3a software (COMSOL AB, Stockholm, Sweden). The diffusion constant of ciprofloxacin was considered to be 6.87 × 10^–6^ cm^2^/s (Stewart, 1996). The model calculation confirmed the formation of a linear concentration gradient within the observation channel (Figure 1C).

**FIGURE 2:**
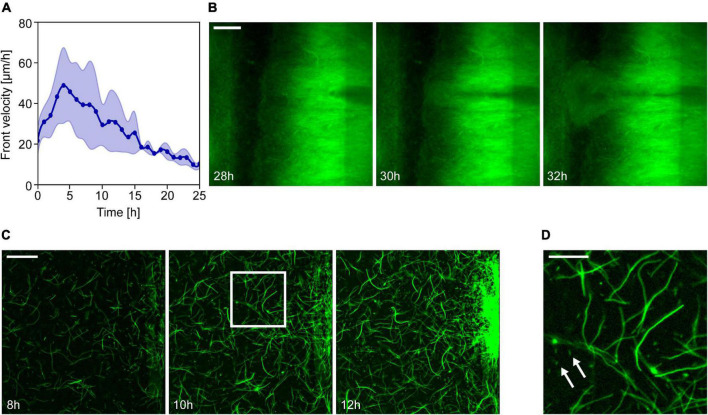
Formation and expansion of resistant *Escherichia coli* populations in ciprofloxacin gradients. **(A)** Changes in the velocity of the propagating front in time. The graph represents the average of the front velocity in experiments with 3× MIC gradient (thick line, *n* = 4) together with the standard deviation. Zero time marks the moment the intense expansion of the population starts. **(B)** Fluorescence images showing the emergence of a less bright but fast-growing subpopulation at a specific location on the low ciprofloxacin side of the channel (scale bar is 200 μm). **(C)** Emergence and growth of a resistant subpopulation on the low antibiotic concentration side of the observation channel. Series of fluorescence images taken at different time points of the experiment (scale bar is 50 μm). **(D)** Zoom-in of the image taken at 10 h to show *E. coli* cells with different morphologies (scale bar is 20 μm). Arrows indicate some normal size rod-shaped cells among filamentous ones.

In each microfluidic experiment, a bacterial suspension was injected into the observation channel yielding a total number of ∼10^5^ cells in the channel. Subsequently, the reservoirs of the device were filled. In the antibiotic gradient experiments, the reservoir on the right side was always filled with antibiotic-free LB medium. The left reservoir was filled with LB medium supplemented with 48 ng/ml (3× the MIC for the wild type strain used in the experiments) or 96 ng/ml (6× MIC) ciprofloxacin (Sigma-Aldrich, St. Louis, MO, United States). In control experiments with no gradient, antibiotic-free LB medium or LB medium supplemented with 48 ng/ml (3× MIC) ciprofloxacin was filled into both reservoirs.

For the duration of the experiments, the microfluidic devices were incubated at 30°C. Besides the control (no gradient) and co-culturing experiments, 20 microfluidic experiments were run. In 16 cases, the devices were continuously monitored by fluorescence time-lapse microscopy. In four cases, fluorescence microscopy imaging was only performed at the end of the experiment.

### Image Acquisition and Image Analysis

Fluorescence time-lapse microscopy was used to monitor the distribution and growth of bacteria within the device. Images were taken every 10 min. The microscope setup contained a Nikon Eclipse Ti-E inverted microscope (Nikon Corporation, Tokyo, Japan), a 10× or 20× Nikon Plan Fluor objective, a GFP fluorescence filter set (49002 filter set, Chroma Technology Corp., Bellows Falls, VT, United States), a Prior Proscan II motorized stage (Prior Scientific Instruments Ltd, Cambridge, United Kingdom), a LUMEN 200 Pro metal arc lamp (Prior Scientific Ltd., United Kingdom) and a cage incubator (Okolab S.r.l., Pozzuoli, Italy). Microscope images were captured by an Andor NEO sCMOS camera (Andor Technology Ltd, Belfast, United Kingdom), and NIS Elements AR software (Nikon Inc., Japan) was used for image acquisition and microscope control.

Image analysis was carried out using ImageJ (Schneider et al., 2012) and MATLAB (Mathworks, Inc., Natick, MA, United States). PIVlab, a time-resolved particle image velocimetry software was used within MATLAB to determine the spread of the resistant population in the channel (Thielicke and Stamhuis, 2014).

### Isolation of Mutants From the Device and Determining Minimal Inhibitory Concentration Values

The following procedure was applied to collect and analyze mutants from the microfluidic experiments.

**FIGURE 3:**
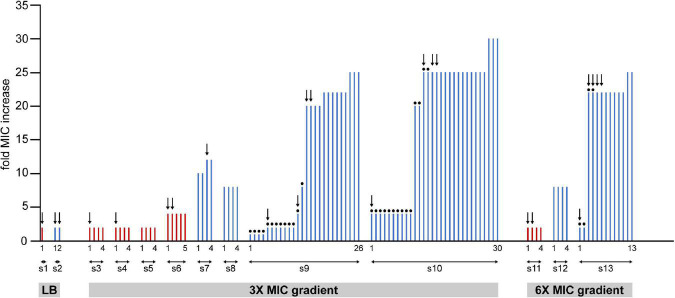
Summary of the measured changes in the MIC values of clones isolated from samples of 13 independent experiments (from s1 to s13). Cells from frozen stocks of samples collected from the microfluidic devices were cultured overnight and spread on agar plates. Bars represent MIC values measured on colonies grown on these plates. Red and blue bars represent samples from 48 to 72 h long experiments, respectively. Dots on top of bars designate colonies grown from cultures that were enriched in LB without ciprofloxacin after cells were picked from the frozen stocks. In other cases (no dots) enrichment was done in LB containing 16 ng/ml (1× MIC) ciprofloxacin. Colonies from each experiment are numbered, the first and the last ones are labeled on the figure.

(1) Out of all 20 microfluidic gradient experiments, 13 devices were broken open 48 h (*n* = 6) or 72 h (*n* = 7) after inoculation to isolate bacterial cells and screen mutations. Bacteria were collected from the open devices by suction with a pipette. The channel area (both the polydimethylsiloxane and the membrane) was washed with 200 μl LB.
(2) Resistant mutants were selected by adding this suspension to culturing tubes containing 3 ml LB medium with 16 ng/ml (1× MIC) ciprofloxacin. After culturing overnight (30°C, 200 rpm), glycerol stocks were prepared from this suspension and were stored at −80°C for later use.
(3) Overnight cultures inoculated from the frozen stocks and grown in LB medium without antibiotics or with 16 ng/ml ciprofloxacin (30°C, 200 rpm) were spread on antibiotic-free LB agar plates.
(4) Colonies from different microfluidic experiments were randomly picked and cultured in antibiotic-free LB medium (30°C, 200 rpm) for MIC determination and subsequent genetic analysis. The MIC values were measured in 96-well plates (Wiegand et al., 2008). Subsequent analysis showed that colonies of mutants with high MIC dominated the plates inoculated from the ciprofloxacin supplemented cultures. In contrast, colonies of lower MIC mutants were found on plates inoculated from antibiotic-free suspensions (Figure 3).

It has been suggested that *in situ* MIC values may depend on the exact conditions (Ersoy et al., 2017). Although in light of this, an assay utilizing our microfluidic platform could be developed, we applied a standard broth dilution MIC measurement scheme to obtain results that can be compared to data in the literature.

### DNA Extraction and Whole Genome Sequencing

Total DNA was extracted by using the Quick-DNA Fungal/Bacterial Microprep Kit (Zymo Research Corp., Irvine, CA, United States) according to the manufacturer’s instructions. Next generation sequencing library preparation has been performed with Nextera XT DNA Library Preparation Kit according to the manufacturer’s protocol. Indexed libraries have been sequenced on Illumina NextSeq 500 sequencer using 2 × 150 PE sequencing (DeltaBio 2000 Ltd., Szeged, Hungary). The sequenced samples included one clone of the ancestral strain and 23 clones isolated from the experiments. The obtained reads were mapped onto the reference genome of *E. coli* W3110. Mutations that appeared in the ancestral strain were discarded.

## Results

We used a microfluidic gradient generator device (Nagy et al., 2015) to study the evolution of antibiotic resistance in motile *E. coli* bacteria exposed to antibiotic gradients in a controlled manner. Figures 1A,B show the schematics of the microfluidic gradient generator device used in our experiments. In this flow-free device, diffusion of antibiotics between two reservoirs through a porous membrane establishes a linear concentration gradient across the width of the observation channel, in which GFP labeled cells of a motile *E. coli* strain (JEK1036) were loaded. To establish an antibiotic gradient, we used a cell division inhibitor, ciprofloxacin (a bactericidal antibiotic). The highest concentration of ciprofloxacin within the device was 3–6-fold the minimal inhibitory concentration (MIC) measured on the ancestral, non-resistant strain which is naive to our device (i.e., it was not cultured in the microfluidic device before). The MIC of this ancestral strain was measured to be 16 ng/ml (Throughout the manuscript when describing the ciprofloxacin concentration in culture media, we use “*n* × MIC” designating concentrations of *n* × 16 ng/ml). In all experiments, ciprofloxacin was loaded into the reservoir on the left side of the microfluidic device, as depicted in Figures 1A,B, so the antibiotic concentration gradient is established with a higher concentration on the left than on the right. Theoretical concentration profiles of ciprofloxacin across the width of the observation channel are presented in Figure 1C. The dynamics of the spatial distribution of the bacterial cells at a population scale was followed by fluorescence time-lapse microscopy.

### Colonization of the Channel by *Escherichia coli* Bacteria in Antibiotic Gradients

In the first few hours after filling up the reservoirs and inoculating the central channel with a homogeneous spatial distribution of cells, bacteria grew mainly at the peripheries of the channel, likely due to higher nutrient supply in these regions coming from the adjacent large reservoirs (see Figures 1D–F). However, later on, growth was limited at higher antibiotic concentration on the left side. Concurrently with this growth inhibition, we observe the formation of highly fluorescent, biofilm-like dense cell assemblages that localize in the region with sublethal antibiotic dose on the right and from which a sharp frontline population starts expanding to the left across the width of the channel (Figures 1D–F). Microscopy images show a quasi-static texture over these biofilms, suggesting the dominance of sessile cells within these structures. Investigating further, we broke open microfluidic devices to harvest the cells from the observation channel. We found macroscopic soft aggregates of cells that could be grabbed with tweezers and pulled out of the channel while cells remained held together by an extracellular matrix.

In the case of gradients with 3× MIC maximal ciprofloxacin concentration, we observe two main patterns of invasion into the high antibiotic zone as shown in Figures 1D,E. One pattern corresponds to the case where a constant spread of the population from the low to the high ciprofloxacin side of the channel can be observed to exhibit a clear, sharp, straight advancing frontline (Figure 1D). Within 12 h, the expanding populations usually reach midway of the channel. The other typical growth pattern is slightly different, as shown in Figure 1E. Here the frontline appears to be more rugged. This ruggedness is shaped by growing streams of bacteria with varying levels of brightness, which is considered as a sign of radiating phenotypic diversity on display. The streams are formed by fast-growing subpopulations which emerge at specific locations on the low ciprofloxacin (right) side of the observation channel and later spread across it at a higher rate than the surrounding cells (see Supplementary Movies 1, 2). In both cases, intense colonization started within the first 10 h of the experiments (Δt = 7.2 ± 3 h; *n* = 5). When applying a steeper gradient, we observe a similar but delayed growth pattern in our microfluidic devices (Figure 1F). In ciprofloxacin gradients with 6× MIC maximal concentration, the rugged frontline begins to invade from left to right later, after approximately 20 h (Δt = 20.5 ± 10.7 h; *n* = 3). Kymographs of representative experiments for both the 3× MIC and the 6× MIC cases are shown in Supplementary Figures 1B,C.

Control experiments were also carried out with no antibiotics (LB medium loaded in both reservoirs of the microfluidic device) and homogeneous distributions of ciprofloxacin (LB medium containing ciprofloxacin loaded in both reservoirs). Kymographs of these experiments are shown in Supplementary Figures 1A,D together with kymographs of the experiments presented in Figures 1D,F. In devices without antibiotics cells grew vigorously all across the observation channel. This demonstrates that our experimental device supports even bacterial growth, which develops into a symmetric spatial distribution. In the case of evenly distributed ciprofloxacin with a concentration of 3× MIC growth was completely inhibited. After about 4 h the fluorescence intensity of cells decreased monotonously, marking the onset of antibiotic induced cell death.

In experiments with antibiotic gradients, the colonization of the channel always involved a sharp leftward propagating population front. We analyzed the velocity of these fronts, and Figure 2A shows these results for 3× MIC gradients. In the early phase of propagation, the front velocity reached an average of 49 ± 22 μm/h (*n* = 4), later decreasing monotonically as cells intruded into areas of higher antibiotic concentration. Finally, the front entirely stopped after reaching the left side of the channel corresponding to the highest ciprofloxacin concentration.

The dominance of sessile cells within the cell assemblage inhabiting our devices resulted in quasi-stationary fluorescence patterns that expanded leftward with the propagation of the frontline. This feature enabled us to perform particle image velocimetry to discover that the expansion velocity was the highest at the front and continuously decreased toward the right side of the observation channel (Supplementary Figure 2). This is in accordance with a scenario of distributed growth within the biofilm and a physically limited expansion that is only possible toward the left. In some cases, we observed the emergence of localized subpopulations (often with altered fluorescence intensity) with considerable growth advantage, shooting through the established population toward the high ciprofloxacin concentration side as shown in Figure 2B. These spatial events of localized growth advantage cause the frontline of the expanding population to become rugged (i.e., Figure 1E). The frequency of these events and the ruggedness of the frontline seems to increase for steeper gradients.

It has been shown before that non-resistant *E. coli* cells exhibit a filamentous morphology even in sub-lethal concentrations of ciprofloxacin (Diver and Wise, 1986; Wojnicz et al., 2007). Furthermore, a previous study linked the emergence of ciprofloxacin resistance to filamentation and a budding phenomenon (Bos et al., 2015). Therefore, we analyzed cell morphology with higher magnification microscopic imaging. We performed this analysis in experiments with 3× MIC maximal ciprofloxacin concentration where the device was inoculated with a low number of cells (∼1,000). In this case, we found a population dynamics similar to those seen in previous experiments, only delayed in time. The intense cell growth on the low antibiotics side did not start until approximately 12 h after inoculation. At that time, local subpopulations began to expand from discrete foci along the right edge of the channel. The biofilm-forming ability of the wild-type strain can explain the formation of these loci (Supplementary Figure 3), which later merge into a larger expanding mass. High magnification images of these regions (Figure 2C) reveal that most cells exhibit an elongated, filamentous shape in response to ciprofloxacin; there are, however, some bacteria with normal rod-shaped morphology as well (shown by arrows in Figure 2D). Although these filamentous cells show less surface attachment (Supplementary Figure 3) and mobility, they formed a dense intertwined multicellular mass that maintained some spatial structure.

### Characterization of Cells Extracted From the Microfluidic Device

To investigate further what changes occur in bacteria that experience the microfluidic environment and the ciprofloxacin gradient, cells were extracted from 13 devices for MIC measurements and whole genome sequencing studies. Figure 3 summarizes the measured MIC changes of individual colonies selected from different experiments (Throughout the manuscript when referring to measured MIC for cells extracted from the device “*n* × MIC change” designates a measured MIC value of *n* × 16 ng/ml). Altogether, 105 colonies from 13 samples (denoted as s1–s13 on Figure 3) were used for MIC measurements. To our surprise, we learned that experiencing the microfluidic devices conferred bacterial cells an increased value of MIC even when not exposed to antibiotics *on-chip*. Furthermore, results show that the MIC change correlates with the duration of exposure to the antibiotic gradient. Samples extracted after 48 h typically exhibit a 2–4× MIC change, while a 1–30× change was measured for samples collected after 72 h. Measured MIC values vary even within samples taken from a single experiment, which demonstrates a compound community development in the microfluidic habitat, giving rise to genetic diversity. In fact, in the case of samples extracted from antibiotic gradients, lower MIC values were only measured when the extracted bacterial culture was regrown in an antibiotic-free medium before plating out on hard agar. Interestingly, as mentioned before, we also found a slight (2×) increase in resistance when bacteria spent 48 or 72 h in the microfluidic device without antibiotics (Figure 3 samples s1, s2). A biofilm assay revealed a diversity of biofilm-forming ability for the isolated mutants (Supplementary Figure 3).

In order to identify possible mutational causes behind the increased MIC levels, we performed whole genome sequencing on 23 colonies picked from different experiments (indicated by arrows in Figure 3). A summary of the most important genes affected is presented in Table 1. More comprehensive lists with detailed results of all the genetic changes found (single nucleotide polymorphisms (SNPs) and insertions/deletions) are shown in Supplementary Tables 1, 2.

**TABLE 1:** Summary of the experimental parameters including the maximum antibiotic concentration in the gradient, incubation time, MIC change, and the relevant genes altered.

Sample	Gradient	Incubation time (h)	Fold MIC increase	Relevant genes
s1/1	–	48	2×	*soxR*
s2/1	–	72	2×	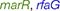
s2/2	–	72	2×	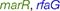
s3/1	3× MIC	48	2×	*mdoG*
s4/1	3× MIC	48	2×	*marR*
s6/1	3× MIC	48	4×	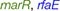
s6/2	3× MIC	48	4×	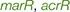
s7/3	3× MIC	72	12×	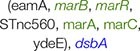
s9/5	3× MIC	72	2×	*ompF*
s9/12	3× MIC	72	4×	*rfaG*
s9/14	3× MIC	72	20×	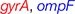
s9/15	3× MIC	72	20×	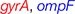
s10/1	3× MIC	72	4×	*dsbA*
s10/13	3× MIC	72	25×	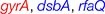
s10/15	3× MIC	72	25×	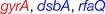
s10/16	3× MIC	72	25×	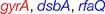
s11/1	6× MIC	48	2×	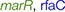
s11/2	6× MIC	48	2×	*galU*
s13/1	6× MIC	72	2×	*ompF*
s13/3	6× MIC	72	22×	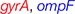
s13/4	6× MIC	72	22×	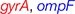
s13/5	6× MIC	72	22×	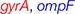
s13/6	6× MIC	72	22×	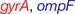

*Color codes of the genes: red—specific function in fluoroquinolone resistance, blue—genes associated with the assembly of the outer membrane, green—genes related to efflux activity.*

In 48 h samples, we found mutations in genes that are not related directly to the mechanism by which ciprofloxacin acts. Instead, these are stress response genes (parts of the *mar* and *sox* operons) are known to be important in general antibiotic and toxin resistance (Miller et al., 1994; Martin et al., 1996; Miller and Sulavik, 1996). They regulate the expression of efflux pumps and various surface structures (e.g., porins). Such mutations were also present in a sample in which cells experienced *on-chip* conditions but were not exposed to antibiotics (sample s1). Among the stress response genes, most frequently *marR* was affected, which is important in the regulation of the expression of the AcrAB efflux pump, which has been reported to play a role in multidrug resistance (Maneewannakul and Levy, 1996; Fernández and Hancock, 2012). In one case (sample s6/2), we found a mutation in *acrR* itself, a direct regulator of *acrAB*. Interestingly, *marR* mutations were also found in 72-h samples cultured in the microfluidic device without antibiotics (sample s2).

Further mutations were found both in 48- and 72-h samples in genes related to the bacterial cell envelope. The *rfa* locus contains the major core-oligosaccharide assembly operons in *E. coli*. Therefore, it has an important role in cell surface hydrophobicity, outer membrane permeability, biofilm formation and aggregation. We found changes (insertion/deletion) in genes mainly involved in the synthesis of the inner core of the lipopolysaccharide (LPS) layer of *E. coli* (*rfaG, rfaE, rfaQ*, and *rfaC*). Besides the *rfa* locus, in the 48-h experiments we occasionally found changes in other genes that are responsible for the proper structure of the cell envelope [*mdoG* (Hanoulle et al., 2004) and *galU* (Shapiro, 1966; Weissborn et al., 1994)].

Data show that the highest jump in antibiotic resistance (>20 fold increase in MIC) is associated with mutations in genes coding one of the target enzymes of ciprofloxacin, namely *gyrA*. We frequently found SNPs causing the amino acid aspartate Asp87 to be changed into glycine (Gly) or tyrosine (Tyr), which are well-known mutations associated with fluoroquinolone resistance (Weigel et al., 1998; Hooper and Jacoby, 2016). Mutations in *gyrA* were always accompanied by mutations in porin protein-coding genes (*ompF*) or other porin-associated genes (*dsbA*). The gene *dsbA* encodes a periplasmic enzyme that catalyzes disulfide bond formation in proteins such as OmpA (Bardwell et al., 1991; Bardwell, 2007), additionally, it is required for flagella and pilus biogenesis (Dailey and Berg, 1993; Alonso-Caballero et al., 2018). These latter mutations by themselves seem to only marginally increase the resistance (2–4×). It is important to note that gyrase and porin-related mutations are only found in 72-h samples. It is interesting to note that we saw a large duplication involving several genes in the mar operon (s7/3) in one instance, which, together with a mutation in *dsbA*, results in a moderate, 12× increase in the MIC in 72 h.

The general pattern we observe here is that several mutations contributed to an initial increase in MIC as cells experience both the stress intrinsic to the microhabitat conditions and the pressure from the antibiotic gradient. A natural question that emerges in the light of our results is, how exaptations (Gould and Vrba, 1982) to tolerate such intrinsic *on-chip* stress, paved the way for further evolution of high antibiotic resistance, and how this is related to the role played by tolerance (Levin-Reisman et al., 2017, 2019).

**FIGURE 4:**
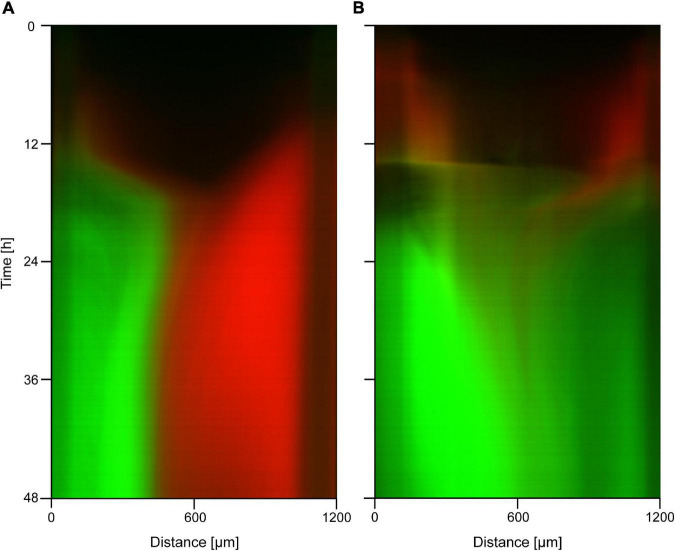
Kymographs of fluorescence microscopy images taken during competition experiments of resistant mutants (s10/16; green color) and the non-resistant ancestor strain (red color) in ciprofloxacin gradients. The applied concentrations are **(A)** 2× MIC on the left side; **(B)** 5× MIC on the left side.

To test if the mutations we observed also endow cells with a competitive advantage in different regions of the gradient, we selected one of the evolved mutant strains extracted from the device (s10/16) and evaluated its growth both as a monoculture and in competition with the wild type strain in the presence of an antibiotic gradient. Results of the monoculture experiments carried out with a maximum concentration of 3× and 6× MIC ciprofloxacin are shown in Supplementary Figures 1E,F. We see that the *on-chip* spatial distribution and growth pattern of this mutant is similar to that of the ancestor strain without antibiotics (Supplementary Figure 1A), showing that ciprofloxacin in such concentrations has little effect on the growth of the evolved strain. In co-culture experiments where the device was inoculated with a mixed culture containing both the GFP-labeled mutant and the RFP-labeled ancestor strain, we observed spatial patterns of community dynamics like the one shown in Figure 4. In the case of a 2× MIC maximal concentration, we observed a compartmentalized coexistence of these strains on a 48-h timescale where the wild-type cells grow in the low antibiotic region while the mutants occupy the high antibiotic zone (Figure 4A). The capacity of the ancestral strain to monopolize the low antibiotic region of the device, suggested by a lack of mixed mutant-ancestor (yellow color), hints at a trade-off between growth rate and antibiotic resistance. In case of 5× MIC maximal concentration, the entire device was dominated by the evolved strain with the population of the ancestor genotype shrinking to near extinction (Figure 4B). In both cases, a seemingly steady-state distribution was reached in about 12 h.

## Discussion

Spatiotemporal structures and heterogeneities are common in natural habitats. Microfluidics offers state-of-the-art tools to engineer synthetic microbial habitats that allow us to mimic the complexity of natural environments (Aleklett et al., 2018; Nagy et al., 2018). In the past decade, a handful of studies applied such technologies to explore the evolution of resistance in the presence of antibiotic gradients (Zhang et al., 2011; Hol et al., 2016; Deng et al., 2019), which yielded seemingly contradicting results.

Hol et al. (2016) used a device with two chambers connected by a narrow, 2 mm long channel. A gradient of kanamycin in LB medium was formed in this channel with a steepness of 25× MIC/mm. Cells from the kanamycin-free chamber formed aggregates and grew and pushed through the channel into the chamber with kanamycin, where they seemed to grow further. However, no MIC increase and no mutations were detected in samples extracted from the device 15–29 h after the invasion of the kanamycin chamber. On one occasion, a 25× MIC increase was found in a sample extracted after 61 h. From these results, the authors conclude that phenotypic tolerance plays a major role in spreading bacterial colonies exposed to antibiotic gradients, at least initially. They suggest that collectively swimming non-resistant bacteria need to exceed a critical population density in order to establish a viable population under lethal antibiotic stress successfully.

Zhang et al. (2011) used a device where the steepness of the ciprofloxacin gradient varied in space in the range of 8–100× MIC/mm in LB medium. They observed a rapid population expansion in about 10 h. Bacteria extracted from the device after approximately 30 h displayed a 12.5× increase in MIC (Bos et al., 2015). Four characteristic mutations in the *gyrA*, *marR* and *rbsA* genes were detected in the resistant strains.

Deng et al. (2019) created a linear ciprofloxacin gradient with a steepness of 1× MIC/mm over an array of interconnected hexagonal microchambers with some resemblance to the device used by Zhang et al. (2011). They imposed an opposite nutrient gradient with M9 medium on the low antibiotics side and LB medium on the high ciprofloxacin side. They measured a slight (2×) increase of MIC on cells extracted from the device after 120 h (Deng et al., 2019).

In our experiments, similarly to Zhang et al. (2011) and Hol et al. (2016), a rapid expansion of the population was seen right after the formation of dense biofilms (in 5–9 h). Although Zhang et al. (2011) considered this expansion a sign of fixation of highly resistant mutants, our results and those by Hol et al. (2016) suggest that phenotypic tolerance due to density-dependent mechanisms is behind this. Indeed, it has been demonstrated that high cell densities involved in swarming and biofilm formation support antibiotic tolerance (Butler et al., 2010). Along with the population expansion, we see the formation of large soft cell assemblages, which may provide some protection against the antibiotics by hindering diffusion. Interestingly, Hol et al. (2016) showed that free-swimming cells could live in the antibiotic zones after successfully invading them. In our experiments, expansion is, therefore, rather due to the protective function of the biofilm as high resistance mutations only appear after the late phase of expansion.

On the other hand, Deng et al. (2019) did not observe such an intense population expansion on the low antibiotics side, which may be due to the fact that they used a nutrient poor medium in these regions. A further aspect to consider here is that they used a non-motile DH5α strain as opposed to the motile W3110 derived strains used in the other studies (including our work) which may also affect the formation of cell aggregates and assemblages (Wood et al., 2006) as well as the ability of cells to explore the antibiotic landscape. Furthermore, they did not observe a high elevation in MIC in 120 h, which suggests that this intense growth of a tolerant population may be a prerequisite for the subsequent appearance of mutants with high MIC (Levin-Reisman et al., 2017).

In the above-mentioned microfluidic experiments with antibiotic gradients (Zhang et al., 2011; Hol et al., 2016; Deng et al., 2019), microfluidic devices with a network of connected chambers were used to culture bacteria. In these devices, a concentration gradient of antibiotics was created by diffusion. It has been suggested that such structures may facilitate the evolution of resistance (Kepler and Perelson, 1998). Compartments with different levels of antibiotics (patchiness) may harbor various mutants with various degrees of resistance at the landscape level. Subsequent mutations, occurring first within local populations living in regions with lower antibiotic concentrations, increase the resistance level and may expand into other antibiotic-rich compartments (Baquero et al., 1998; Hermsen et al., 2012). This is in accordance with the concept of selection amplifiers (Tkadlec et al., 2021), structures in which antibiotic-resistant variants enrich.

Although our microfluidic device is spatially distributed, it lacks such an explicit spatial structure on the microscopic scale and is considered a continuous habitat. Still a population with a self-imposed spatial structure emerged in our device due to the organization of cells into biofilms. This is demonstrated by the emergence of patterns due to streams of cells with various fluorescence intensity (Figures 1D–F).

A similar self-organized spatial population structure is observed for large colonies growing on agar gel landscapes (Baym et al., 2016). Such emergent spatial population structures seem to be sufficient for the formation of selection amplifiers; however, in our case, they originate from endogenous dynamics of the population rather than the geometrical fragmentation implicit in the spatial structure of the habitat [as in the case of Zhang et al. (2011)]. The fact that the evolved highly resistant strains presented here carry similar mutations to those seen by Zhang et al. (2011) supports this idea.

As opposed to a homogeneous culture (e.g., a stirred flask) with a global competition between cells/mutants, populations with spatial structure exhibit a more localized competition pattern. Emergent mutants contend mainly against their neighbors for resources, and their interactions with distant individuals are limited. Therefore, beneficial mutations at various locations may emerge, persist and avoid direct competition. This increases genetic diversity and reduces the effects of clonal interference, which may explain the relatively quick increase in resistance seen in our experiments and those by Zhang et al. (2011).

One striking result in our experiments is that we see similar mutations (in the *marR* and *rfaG* genes) and a 2–4× increase in MIC in cells that inhabit our devices in the absence of antibiotics and those experiencing an antibiotic gradient for 48 h. It seems that these mutations were likely induced by the stress imposed by the microfluidic environment itself (e.g., nutrient limitation) and yielded an incidental MIC increase. MarR is important in the regulation of the expression of the ArcAB efflux pump subunit, thus having a role in multidrug resistance (Fernández and Hancock, 2012). The *rfa* operon is involved in the lipopolysaccharide biosynthesis pathway, and *rfa* mutations can change the permeability of the outer membrane and decrease the expression of *ompF* and *ompC* (Schnaitman and Klena, 1993; Fralick and Burns-Keliher, 1994). The pleiotropic nature of these genes makes it possible that the microfluidic habitat induced mutations act as exaptations (Gould and Vrba, 1982). In other words, they emerge due to the constricted environment, but later prove useful when cells adapt to high antibiotic regions in the channel. The importance of exaptations in the evolution of antibiotic resistance has been suggested before (Martinez and Baquero, 2009; Martinez et al., 2009). Our results demonstrate that exaptations may be most important in the early steps of evolutionary processes responsible for the emergence of resistance. Similarly, habitat (device) induced mutations may be behind the slight MIC increase that Deng et al. (2019) observed in their microfluidic experiments. However, as discussed above, the possible nutrient limitation prevented the formation of population expansions that seem to be necessary for the high MIC increase. Interestingly, the above-mentioned mutations were not found in samples exposed to antibiotic gradients for 72 h, which might hint at the existence of complex genomic changes during the pathway of evolving resistance. On the other hand, adaptation on the phenotypic level as well as mutations yielding a slight increase in MIC may be necessary for the later emergence of highly resistant mutants, ensuring the survival of the population for a sufficient length of time (Bernier and Surette, 2013). The fact that we only see highly resistant mutants in the 72-h samples, which are preceded by mutants with lower MIC, supports this idea.

## Conclusion

We have used a gradient generator microfluidic device to study the emergence of tolerant and resistant bacteria in ciprofloxacin concentration gradients. Our results help resolve the seeming contradiction between the various outcomes of previous microfluidic experiments. Moreover, they also cast light on some of the fundamental evolutionary processes taking place.

In 5–9 h after inoculating the device with a non-resistant ancestral *E. coli* population, we observed biofilm formation on the gradient’s low antibiotic region which later expanded into the high antibiotic region. In the context of existing literature, our results suggest that this process is necessary for the later emergence of highly resistant mutants. We propose that in the absence of an explicit spatial structure of the habitat, biofilm formation may lead to an endogenously generated spatial structure within the population which could function as a selection amplifier and aid the spread of beneficial mutants.

Interestingly, we found that, even without any exposure to antibiotics, the microfluidic environment itself induces mutations that result in a slight increase of MIC. These mutations are beneficial and selected for in those cells that get exposed to antibiotics. Such shifts in function suggest that the process of exaptation may occur in our experimental scenario.

## Data Availability Statement

The data generated in this study are available from the corresponding author upon reasonable request. The raw sequences are available at the European Nucleotide Archive (ENA) database under the study number PRJEB47179.

## Author Contributions

KN designed and performed the experiments, participated in the data analysis, and wrote the first draft of the manuscript. BD and OH contributed to the microfluidic device fabrication and running experiments. LD contributed to the data analysis. ÁÁ and EC helped in performing the MIC measurements. MW, JN, and JK contributed to interpreting the data and drafting the manuscript. PG planned and supervised the study, wrote and edited the manuscript. All authors reviewed and approved the manuscript.

## Conflict of Interest

The authors declare that the research was conducted in the absence of any commercial or financial relationships that could be construed as a potential conflict of interest.

## Publisher’s Note

All claims expressed in this article are solely those of the authors and do not necessarily represent those of their affiliated organizations, or those of the publisher, the editors and the reviewers. Any product that may be evaluated in this article, or claim that may be made by its manufacturer, is not guaranteed or endorsed by the publisher.
