# Supplementary material for: Emergence of Resistant Escherichia coli Mutants in Microfluidic On-Chip Antibiotic Gradients
Source: Front Microbiol. 2022 Mar 22;13:820738. doi: 10.3389/fmicb.2022.820738 (PMC8981919; doi:10.3389/fmicb.2022.820738)
Supplement: Supplementary file 1 [file Data_Sheet_1.pdf]

Supplementary Material

CONTENT:
Supplementary Figures 1-3
Supplementary Tables 1-2
Captions for Supplementary Movies 1-2

Other online supplementary material for this manuscript includes:
Supplementary Movies 1-2

Supplementary Figures and Tables:

Supplementary Figure 1. Kymographs showing the distribution of motile, wild-type *E. coli* (JEK1036 strain) in the microfluidic device in different experiments. Fluorescent images were taken every 10 minutes. Color bars indicate the average fluorescence intensity values (a.u.). A) *E. coli* cultured in the device with no antibiotics. B) *E. coli* in ciprofloxacin gradient with a maximum concentration of 3×MIC loaded into the left reservoir. C) *E. coli* in ciprofloxacin gradient with a maximum concentration of 6×MIC loaded into the left reservoir. D) *E. coli* cultured in the device with homogeneously distributed 3×MIC ciprofloxacin. E) Ciprofloxacin resistant mutant *E. coli* strain in ciprofloxacin gradient with a maximum concentration of 3×MIC loaded into the left reservoir. F) Ciprofloxacin resistant mutant *E. coli* strain (s10/16) in ciprofloxacin gradient with a maximum concentration of 6×MIC loaded into the left reservoir.

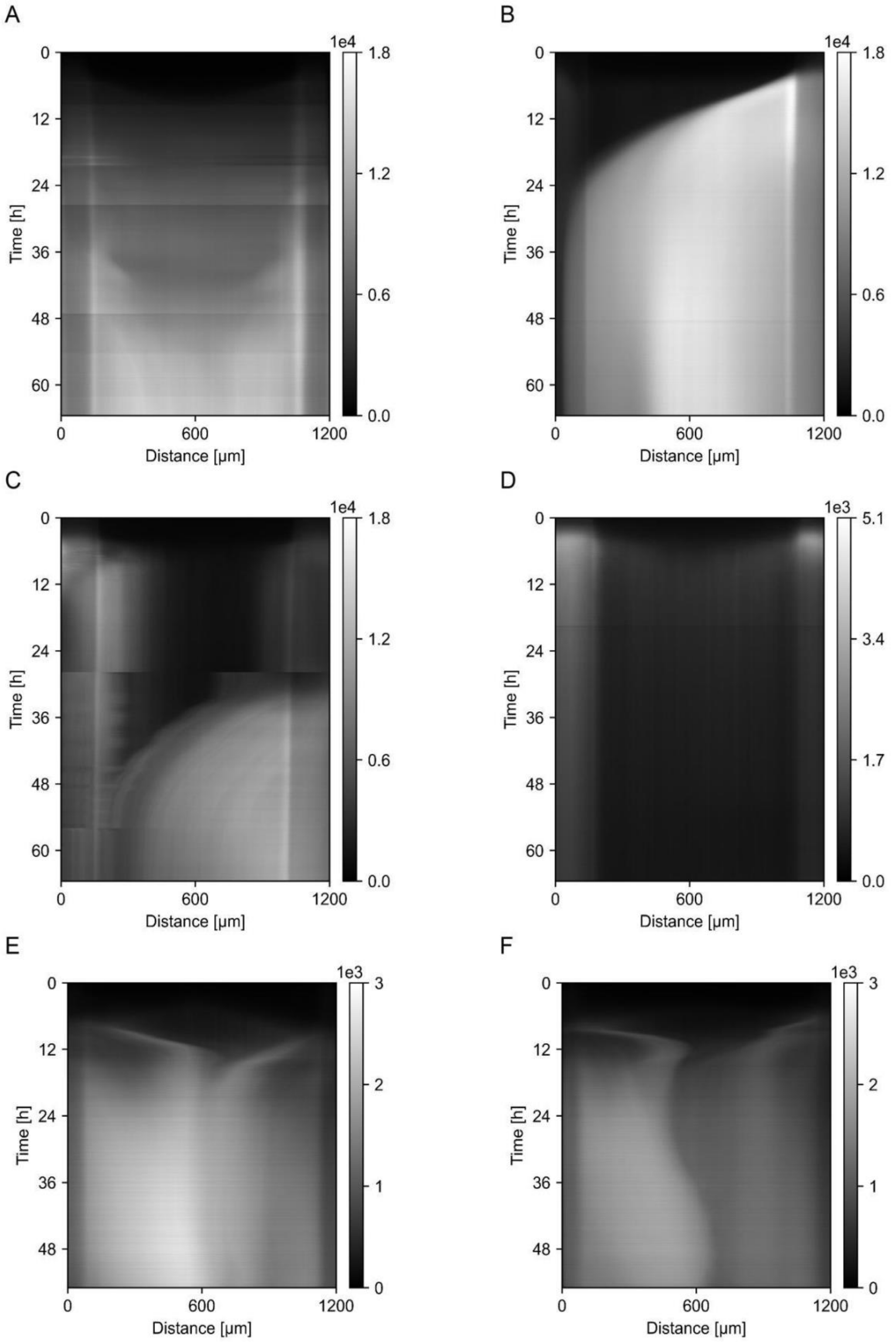
A
B
C
D
E
F
Time [h]
Distance [μm]
0
12
24
36
48
60
600
1200
1e4
1.8
1.2
0.6
0.0
1e3
5.1
3.4
1.7
3
2
1

Supplementary Figure 2. Particle Image Velocimetry analysis of microscopy images.
A) Microscopy images with velocity vectors at three different time points (16h, 20h, 24h) of an experiment to show the propagation of the bacterial population across the observation channel from the low ciprofloxacin side towards the higher concentration regions. The experiment was carried out with a 3×MIC maximum concentration of ciprofloxacin loaded into the left reservoir. The scale bar is 200 µm. B) Velocity profiles across the observation channel at specific time points.

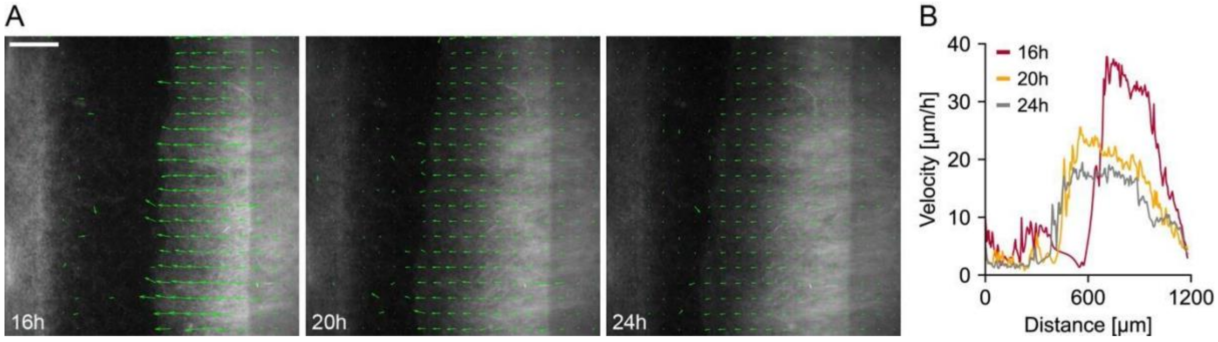

Supplementary Figure 3. Biofilm forming ability of the ancestral and mutant strains evolved in the microfluidic device in the presence of a ciprofloxacin gradient obtained by the microtiter plate biofilm assay (based on crystal violet staining). The assay was performed in a 96-well plate in which cells were incubated for 24 hours at 30 °C without antibiotics and with 10 ng/ml and 20 ng/ml ciprofloxacin (0.63×MIC and 1.25×MIC of the ancestral strain, respectively). Three replicates were made of each sample.

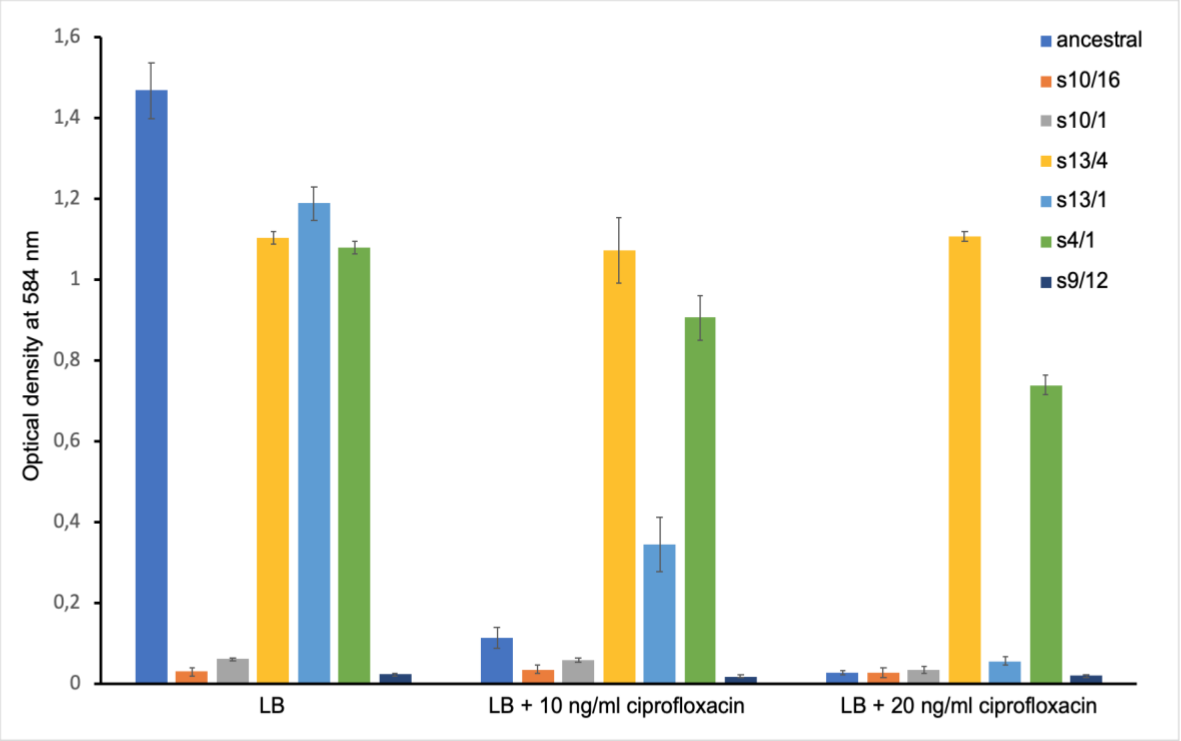

Supplementary Table 1. Table showing all single nucleotide polymorphisms and small insertions/deletions detected compared to the reference genome in the 23 sequenced samples extracted from the microfluidic device.

Position	Type	Reference	Alt allele	Gene	CDS/genomic	Protein	s1/1	s2/1	s2/2	s3/1	s4/1	s6/1	s6/2	s7/3	s9/5	s9/12	s9/14	s9/15	s10/1	s10/13	s10/15	s10/16	s11/1	s11/2	s13/1	s13/3	s13/4	s13/5	s13/6
225813	DEL	GACGTGCTAA	G	rrlH	n.56_64delACGTGCTAA																	x							
406748	SNP	C	T	aroM	c.97C>T	p.His33Tyr					x																		
891635	SNP	C	G	nfsA	c.30C>G	p.Gly10Gly																	x						
1213665	IN	C	CC	elbA-ycgX	n.1213665_1213666insC									x											x				
1276448	SNP	A	C	ychP	c.947A>C	p.Asp316Ala																			x				
1382000	SNP	A	C	ycjU	c.139A>C	p.Ile47Leu															x								
1620952	DEL	CGGCACAGTTTAAGG	C	marR	c.120_133delGGCACAGTTTAAGG	p.Gln42fs		x	x																				
1621018	IN	A	AA	marR	c.185dupA	p.Val63fs																	x						
1621072	DEL	GTAAAGGCTGGGTG	G	marR	c.240_252delTAAAGGCTGGGTG	p.Cys80fs						x	x																
1621193	DEL	CC	C	marR	c.361delC	p.Gln121fs					x																		
1622191	SNP	C	G	eamA	c.661G>C	p.Ala221Pro												x											
2343831	SNP	T	C	gyrA	c.260A>G	p.Asp87Gly														x	x	x							
2343832	SNP	C	A	gyrA	c.259G>T	p.Asp87Tyr											x	x								x	x	x	x
2477447	IN	C	CTGCC	yfdL	c.61_62insGGCA	p.His22fs																x							
3194840	DEL	CGTAGCGC	C	rfaE	c.563_569delGCGCTAC	p.Ala189fs						x																	
3367311	SNP	C	A	yhcF	c.531C>A	p.Thr177Thr																x							
4281426	SNP	G	C	soxR	c.368G>C	p.Gly123Ala	x																						
4640608	SNP	C	A	creA	c.408C>A	p.Asp136Glu									x														

Supplementary Table 2. Table showing all large insertions/deletions detected compared to the reference genome in the 23 sequenced samples extracted from the microfluidic device.

Start	End	Genes	Type	s1/1	s2/1	s2/2	s3/1	s4/1	s6/1	s6/2	s7/3	s9/5	s9/12	s9/14	s9/15	s10/1	s10/13	s10/15	s10/16	s11/1	s11/2	s13/1	s13/3	s13/4	s13/5	s13/6
353244	353254	prpE	INS																				x			
485098	485101	acrR	INS							x																
986618	986627	ompF	INS									x		x	x							x	x	x	x	x
987437	987447	-	INS										x													
987492	987502	-	INS					x																		
1111314	1111325	mdoG	INS				x																			
1293677	1293699	galU	INS																		x					
1294222	1294231	hns	INS	x																						
1574068	1574075	-	INS								x															
1620547	1624458	eamA, marB, marR, STnc560, marA, marC, ydeE	DUP								x															
3187732	3187735	yqiG	INS														x	x	x							
3419160	3419169	yhdW	INS													x										
3523484	3523497	yiiS	INS	x																						
3592940	3592952	dsbA	INS								x					x	x	x	x							
3833226	3833234	rfaQ	INS														x	x	x							
3833957	3833970	rfaG	INS										x													
3833994	3834004	rfaG	INS		x	x																				
3843646	3843649	rfaC	INS																	x						

Captions for the additional online supplementary material:

Supplementary Movie 1. A time-lapse fluorescence microscopy recording showing one of the characteristic patterns we observed for an *E. coli* population in a ciprofloxacin gradient. The maximum concentration of antibiotics (3×MIC) is on the left side. A constant spread of the population from the low cipro (right) side to the high cipro (left) side of the channel takes place with a clear, sharp, and straight advancing frontline

Supplementary Movie 2. A time-lapse fluorescence microscopy recording showing another typical growth pattern we observed for an *E. coli* population in ciprofloxacin gradient. The maximum concentration of antibiotics (3×MIC) is on the left side. The population spreads with a less sharp, rugged frontline. This ruggedness is shaped by growing streams of bacteria with different fluorescence brightness. The streams are formed by fast-growing subpopulations which emerge at specific locations on the low ciprofloxacin (right) side of the observation channel and later spread across it at a higher rate than the surrounding cells.
